# The Effect of Cellulose Nanocrystal Coatings on the Glass Fiber–Epoxy Interphase

**DOI:** 10.3390/ma12121951

**Published:** 2019-06-17

**Authors:** Joyanta Goswami, Ejaz Haque, Douglas M. Fox, Jeffrey W. Gilman, Gale A. Holmes, Robert J. Moon, Kyriaki Kalaitzidou

**Affiliations:** 1 George W. Woodruff School of Mechanical Engineering, Georgia Institute of Technology, Atlanta, GA 30332, USA; jgoswami6@gatech.edu; 2 School of Material Science and Engineering, Georgia Institute of Technology, Atlanta, GA 30332, USA; ehaque3@gatech.edu; 3 Department of Chemistry, American University, Washington, DC 20016, USA; dfox@american.edu; 4 Materials Science and Engineering Division, Material Measurement Laboratory, National Institute of Standards and Technology, Gaithersburg, MD 20899, USA; jeffrey.gilman@nist.gov (J.W.G.); gale.holmes@nist.gov (G.A.H.); 5 The Forest Products Laboratory, U.S. Forest Services, Madison, WI 53726, USA; robertmoon@fs.fed.us

**Keywords:** cellulose nanocrystal, coating, glass fiber, interphase, shear strength, fracture

## Abstract

This study focuses on understanding the effect of cellulose nanocrystals (CNCs) on glass fiber/epoxy interfacial interactions. The glass fibers (GF) were coated with solutions containing cellulose nanomaterial. The parameters that were investigated were the CNC surface chemistry, concentration, and dispersing medium, i.e., aqueous solution only versus emulsions. To determine the effect of the CNC coatings on the interfacial adhesion, specimens of a single GF in an epoxy matrix were prepared for GF coating by varying the coating formulations. The interfacial shear stress (IFSS) was determined by the single fiber fragmentation test (SFFT). Following the SFFT, the samples were investigated by cross-polarized microscopy in order to understand the fracture modes which are related to the nature of the interphase. According to the SFFT data and photoelastic fracture patterns, both the emulsion and aqueous coatings containing cellulose nanocrystals functionalized with methyl(triphenyl) phosphonium (CNCPh) improve the IFSS in comparison to coated GFs without CNCs.

## 1. Introduction

Fiber-reinforced polymer (FRP) composites are frontrunners in structural polymer applications. The expanding areas of FRP structural applications include mortar reinforcement in masonry [1], restoring seismic damaged buildings and historical building preservation [2], bridge girders [3], energy-efficient sandwich glass panels [4], and lightweight automotive components [5]. An essential attribute of FRP composites is their load transfer ability across the fiber–polymer interphase, a region extending from the interface of the fiber toward the polymer matrix that spans from tens of nanometers to several hundred nanometers [6,7]. Since the interphase dictates the composite properties, exhibiting control over the fiber surface properties during the manufacturing process is a key goal.

The contribution of the interphase on fiber/matrix adhesion is physicochemical in nature and involves surface texture-induced friction, intermolecular interactions, chemical coupling, surface crystallization, and phase separation. The prominent routes to engineering the interphase are the (i) application of sizing on the fiber, (ii) introducing functional groups on the fiber surface, (iii) chemical modification of the polymer, and (iv) coating the fibers with nanomaterials. The sizing on the fibers is an integral part of glass fiber (GF) manufacturing, with its role going beyond the enhancement of the interphase [8]. It consists of an optimized formulation of polymer, surfactants, silanes, lubricants, and antistatic agents. The effect of the sizing formulation on fiber/matrix adhesion has been extensively investigated, including the incorporation of nanomaterials, such as carbon nanotubes, in the formulations [9,10,11,12]. Other nanomaterials that have been introduced on the surface of the fiber are graphene nanoplatelets and graphene oxides [13,14] and colloidal silica [15]. This study focuses on cellulose nanocrystals (CNCs), which have successfully been used as polymer reinforcement nanomaterials but have not been systematically investigated with regard to their role in engineering the fiber/polymer interphase.

CNCs are cellulose-based spindle-shaped nanoparticles (between 3 and 20 nm in width and between 50 and 500 nm in length) that can be extracted from trees and plants by acid hydrolyses [16,17]. They are viable candidates as additives to alter the interphase properties because of their many useful attributes. These particles have low densities (1.6 g/cm^3^) comparable to those of polyparaphenylene terephthalamide (Kevlar, 1.4 g/cm^3^) and carbon fiber (1.8 g/cm^3^) but far lower than that of steel (7.8 g/cm^3^) [16,18]. CNC mechanical properties (e.g., tensile strength between 7.5 and 7.7 GPa and elastic modulus between 110 and 220 GPa) [16] are also comparable to those of Kevlar (tensile strength of 3.5 GPa, elastic modulus between 124 and 130 GPa) [19] and carbon fiber (tensile strength between 1.5 and 5.5 GPa, elastic modulus between 150 and 500 GPa) [19]. CNCs also have a high surface area (≈13 m^2^/g) [20] with accessible hydroxyl side groups that can be modified to add the desired chemical functionality [16,18,21]. Additionally, since the global production volumes of CNCs are increasing while costs are decreasing, these materials are becoming more viable for industrial applications [18,22].

Common to many nanomaterials, the limiting factor in utilizing the unique properties of CNCs as reinforcements in polymers is their agglomeration and poor dispersion within the said polymers. This challenge has been previously addressed by modifying the CNC surface and using complex wet chemistry routes and solvent exchange methods [23,24]. However, some of these methods are not industrially viable due to their limited scalability. Nonetheless, CNCs have successfully been used in the formulations of coatings for fibers, resulting in an enhancement of the properties of the corresponding FRP composites [25,26].

In this work, the effect of CNCs on the GF–epoxy interphase was investigated as a function of the CNC concentration in solution, CNC surface functionality, and dispersion medium used to introduce the CNCs into the fiber coating. The surface functionalities studied included sulfate half esters, carboxylate groups, and counter ion exchanges. The methods studied were dip coating of GF in aqueous dispersions, emulsions, and commercial sizing mixtures that contain CNCs. The effect of CNCs on GF–epoxy adhesion was primarily assessed by the single fiber fragmentation test (SFFT). This study provides a basic understanding of the effect of CNCs on GF–epoxy interactions and guidelines for the selection of the optimal CNC-based sizing of GFs for composites with enhanced properties.

## 2. Materials and Methods 

### 2.1. General Information

Low viscosity epoxy 635 (70% to 90% 4,4′-Isopropylidenediphenol-Epichlorohydrin Copolymer and 20% to 25% Oxirane, Mono[(C12-14-alkyloxy) methyl] derivatives) and hardener 556 (40% to 55% Polyetheramine, 30% to 45% nonylphenol, 10% to 30% 2-piperazin-1ylethylamine) used in the epoxy curing mixture preparation in a ratio of 2:1 epoxy:hardener were purchased from US Composites, West Palm Beach, FL, USA. Multiend roving glass fibers ME1510 (TEX 4800, average single fiber diameter 10 μm) with silane sizing and compatibility for epoxy matrices were obtained from Owens Corning, Oak Brook, IL, USA.

The aqueous dispersion of 11.9% cellulose nanocrystals, derived from wood by sulfuric acid hydrolysis (CNCS) and manufactured by Forest Product Laboratory (FPL), was purchased from the University of Maine, Orono, ME, USA. The length and width of CNCS were 134 nm (±54 nm) and 7 nm (±2 nm), respectively, with 1 standard deviation in the parentheses as determined by Reid et al. [27]. A 4% aqueous dispersion of cellulose nanocrystals with methyl(triphenyl) phosphonium (CNCPh) functionality prepared by an ion exchange process as described previously [24] was also used in this study. The average length and diameter of the CNCPh were 130 nm and 6 nm, respectively. The CNC dispersions were also dispersed in commercial fiber sizing EP871, a nonionic epoxy emulsion obtained from Michelman, Inc., Cincinnati, OH, USA. Finally, a triblock co-polymer, Pluronic P123 (M_n_ = 5800), and a 0.1% Poly-L-lysine (PLL) aqueous solution, both purchased from Sigma-Aldrich, St. Louis, MO, USA, were also used as dispersing media for CNCs.

### 2.2. CNC-Dispersion and Emulsion Preparation

The preparation of CNC coating formulations involved dilution of the as-received CNC dispersions. The diluted CNC dispersions were magnetically stirred for 10 min at 52.36 rad/s, followed by sonication using a Qsonica 500 ultra sonicator with a probe diameter of 13 mm. The sonication procedure involved 5 min of sonication at a 60% amplitude with a 5 s pulse on, 3 s pulse off cycle in an ice-water mixture.

Two types of CNC-emulsions were also used as a coating for GF: i) A laboratory-based emulsion prepared with the same epoxy as the matrix, and ii) a commercial epoxy-based sizing emulsion. The laboratory-based emulsion was prepared by magnetically stirring sonicated aqueous CNC dispersions with Pluronic P123 for 1 h in an ice-water bath, followed by the addition of thin epoxy 635 (triblock-co-polymer: epoxy = 1:4) and further stirring for 30 min. Commercial-based sizing emulsions were prepared by adding an appropriate commercial emulsion, EP871, to sonicated CNC dispersions and stirring magnetically at 52.36 rad/s for 30 min. The control epoxy-based emulsion was prepared similarly using distilled water instead of a CNC dispersion.

### 2.3. Addition of CNCs on the GF Surface

The addition of CNCs to the GF surface was completed by dipping GF into various CNC dispersions or emulsions. The CNC dispersion/emulsion parameters were: (i) Concentration (e.g., 0.1%, 0.5%, 1%, or 2%); (ii) surface functionalization (e.g., CNCPh, CNCS); (iii) addition of dispersing agent (e.g., PLL); and iv) emulsion (e.g., epoxy 635, EP871). The coating process involved mounting 8 or 9 single glass fibers, obtained by hand from GF rovings, in tension along the length of a rectangular frame. The frame was submerged horizontally in 120 mL of coating dispersion for 5 min, dried for 8 to 12 h at ambient temperature, then dried again at 100 °C for 20 min. In the case of the PLL/CNC coating, the single fibers were submerged in 0.01% PLL at pH = 8 for 30 min and dried for 2 h under ambient conditions before dip coating in the CNC dispersion. It should be noted that in no case was the pre-existing silane sizing removed from the fiber surface prior to coating with CNC, as doing so would result in fiber degradation through the introduction of surface defects.

The coating code name and coating dispersions used in the study are listed in Table 1 and Table 2. The first letter in the coating formulation code represents the fiber material (G, glass), the second letter indicates dispersion media type, and the last letter with Roman numerals distinguishes between the types of CNC used. A number prior to a letter indicates the concentrations of media or CNCs.

### 2.4. Fiber and Composite Imaging

The morphology/surface of the coated glass fibers was examined by scanning electron microscopy (SEM) using a Hitachi SU8230 (Hitachi, Tokyo, Japan). Single glass fibers were mounted on carbon tape, sputter coated with gold, and probed under SEM at an electron acceleration voltage of 5 kV. The fragmentation type and fragment length of fibers inside the epoxy dog bone tensile coupons after the single fiber fragmentation tests (SFFT) were analyzed and measured by cross-polarized optical microscopy using a Leica DM 2500 (Leica Microsystems, Wetzlar, Germany). The process for curing these coupons is illustrated in Section 2.5.

### 2.5. Characterization of Interfacial Shear Strength (IFSS) 

**Figure 1:**
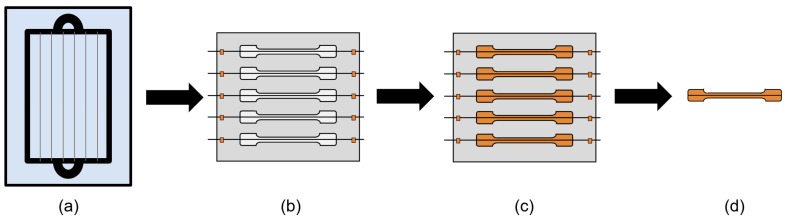
Preparation of SFFT coupons. (**a**) Individual as-received GF mounted on a rectangular frame, subsequently immersed in the desired sizing solution (first immersed in PLL in the case of PLL coatings). (**b**) Dried fibers are removed from the frame and mounted on the mold using heat resistant tape to maintain fiber tension. (**c**) Resin/hardener mixture is poured into mold cavities. (**d**) Cured samples are removed using push pins on the underside of the mold.

The interfacial shear strength was determined experimentally based on SFFT. The testing coupons consisted of a single fiber embedded in epoxy. Specifically, a single glass fiber was mounted using tape on both ends of a dog bone shaped mold to maintain sufficient tension in the fiber to compensate for thermal compression introduced during the curing stage. The epoxy curing mixture was then poured into the mold cavity. For each coating formulation, 5 to 7 single fiber/epoxy tensile specimens were prepared, cured at 100 °C for 1 h then 120 °C for 2 h, and tested. This specimen preparation process is illustrated in Figure 1.

The specimens were subjected to tensile testing using an Instron 33R 4466 with a load cell of 500 N and extension rate of 1 mm/min. As the externally applied tensile load is increased, the stress is transferred through shear forces from the polymer to the fiber. During this loading process, the fiber continues breaking until the length of the fragments reaches a critical value below which no more fiber breaks occur. The specimens from the tensile test were examined under a cross-polarized microscope to determine the number and lengths of the fiber fragments. From these data, the interfacial shear strength (IFSS, τ) is estimated using an equation that has the general form delineated on the left-side of Equation (1), where $d_{f}$ is the fiber diameter, $f\left\{l_{c}\right\}$ is an explicit function of the critical transfer length, and $l_{c}$, and $\sigma_{f}\left(l_{c}\right)$ is the strength of the fiber at $l_{c}$:

(1) $$\frac{d_{f}}{2}f\left\{l_{c}\right\}\sigma_{f}\left(l_{c}\right)=\tau \approx \frac{d_{f}\sigma_{f}\left(l_{c}\right)}{2l_{c}}=\frac{d_{f}K^{\prime }\sigma_{f}\left(l_{c}\right)}{2\bar{l_{s}}}.$$

It is well known that FRP composites do not generally conform to the elastic–plastic matrix assumption used to develop the Kelly–Tyson (K–T) model for determining IFSS in metal matrix composites [28,29]. Notwithstanding, the K–T model is typically used to approximate the IFSS in FRP composites by taking $f\left\{l_{c}\right\}=1/l_{c}$ (see right side of Equation (1)) with the following expression used for the critical transfer length: $l_{c}=\bar{l_{s}}/K’$, where K’ is a constant that varies between 0.668 and 0.75 [30]. It follows that τ is a function of the average fragment length at saturation, $\bar{l_{s}}$. Because there is no accepted method for determining $\sigma_{f}\left(l_{c}\right)$ [30,31], estimates of τ can vary widely. For this study, $\bar{l_{s}}$ was used as an acceptable surrogate for τ since it depends explicitly on this parameter. IFSS comparisons were thus made on the basis of $\bar{l_{s}}$ and the corresponding number of fiber fragments for each sample.

Within this framework, the SFFT specimens of different coating formulations were initially tensile tested successively at 3, 4, and 5 mm extensions to determine saturation—tensile loading after the fiber fragmentation process ceases. The tensile tests for GF as-received, GF coated in aqueous only systems, and GF coated in laboratory emulsions were terminated at an extension of 3 mm where saturation in fiber fragmentation was reached. For the commercial emulsion coating systems, the saturation was instead reached at the 4 mm extension, and samples were tested to this saturation limit.

### 2.6. CNC–Fiber Interactions 

Coating formulations and coated glass fibers were probed by a Fourier Transform Infrared (FTIR) spectrophotometer (Nicolet iS5, Thermo Fischer Scientific, Waltham, MA, USA) equipped with a diamond crystal attenuated-total-reflection (ATR) probe. Absorption spectra were collected from a total of 64 scans in the range of 400 to 3600 cm^−1^ with a data spacing of 0.241 cm^−1^. FTIR was conducted on GF coated by dip coating 200 mg of chopped fibers in 10 mL of coating dispersion in a 20 mL vial for 5 min. The wet fibers were dried under ambient conditions for 12 h, followed by oven drying at 80 °C for 1 h. The coating dispersions were characterized with FTIR by forming films or pastes by drying dispersions under ambient conditions.

## 3. Results

### 3.1. Probing the Coatings on GF Using FTIR

**Figure 2:**
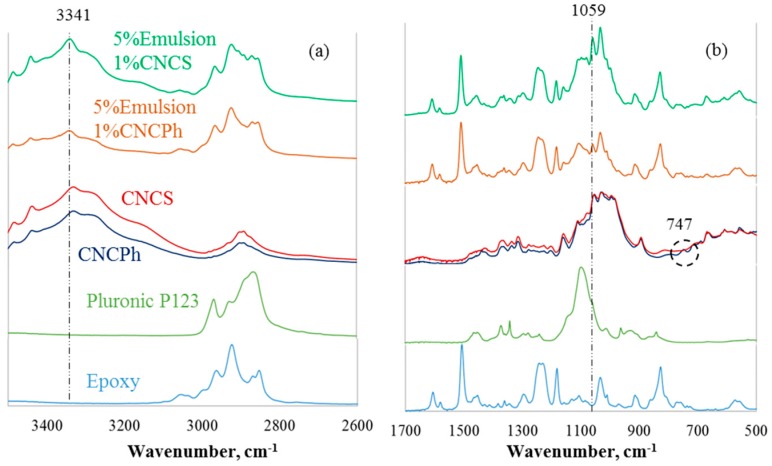
FTIR spectra (**a**) 3500 to 2700 cm^−1^, (**b**) 1800 cm^−1^ to 500 cm^−1^ of CNC films and dried dispersions.

To investigate the presence of the CNCs and the emulsion components on GF, the FTIR spectra of CNC films, CNC-emulsion dried dispersions, and coated GF were measured. The spectra for CNC films and CNC-emulsion dried dispersions is provided in Figure 2. The CNCS and CNCPh exhibited similar peaks between 3500 to 3300 cm^−1^ and 2910 to 2880 cm^−1^, which are characteristic stretching vibrations of O-H and C-H of pyranose rings, respectively [32,33]. The absorption bands in the range of 1160 to 830 cm^−1^ are attributed to stretching vibrations of C-O and C-O-C and rocking C-H vibrations of the pyranose ring [32,33]. The peak differentiating CNCPh and CNCS is at 747 cm^−1^, circled in Figure 2b, which represents the C-H bending vibration of the benzene ring of the phenyl group present in CNCPh. The FTIR spectrum of the triblock-copolymer and the epoxy present in the laboratory emulsion coatings are also shown for reference; no absorption was observed for the emulsion components between 3500 cm^−1^ to 3300 cm^−1^.

**Figure 3:**
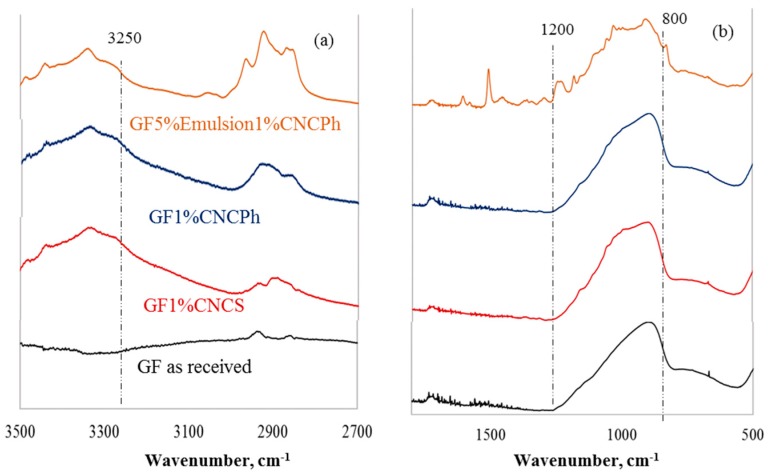
FTIR spectra (**a**) 3500 cm^−1^ to 2700 cm^−1^, (**b**) 1800 cm^−1^ to 500 cm^−1^ of coated GF.

The FTIR spectra of the as-received GF and the fibers coated with aqueous CNC and CNC-emulsion are displayed in Figure 3. The peak at 906 cm^−1^ and the broader peak in the range of 700 to 800 cm^−1^ in the spectra of the as-received GF represent SiO_2_ vibrations in combination with other inorganic oxides [34]. The presence of CNCs in the aqueous CNC coated GF is evident from the broadening of the glass fiber peak in the range of 800 to 1200 cm^−1^ with small peaks, the presence of bands between 3250 to 3500 cm^−1^ and 2810 to 2970 cm^−1^, which were not present in the control spectra (as received GF), and a relatively higher intensity of the peak in the range of 2810 to 2970 cm^−1^. Similarly, the 5% emulsion and 1% CNCPh coating showed the characteristic epoxy and CNCPh bands. The pristine CNCS and CNCPh peaks at 3331 and 1054 cm^−1^ shifted to 3341 and 1059 cm^−1^, respectively, in the emulsion. The 3341 cm^−1^ peak shift for the CNC types was detected for the GF with CNC aqueous dispersion coatings (Ga1CI and Ga1CII) and for the CNCPh-emulsion coating (G5e1CII).

### 3.2. Coating Characterization by SEM and TGA

**Figure 4:**
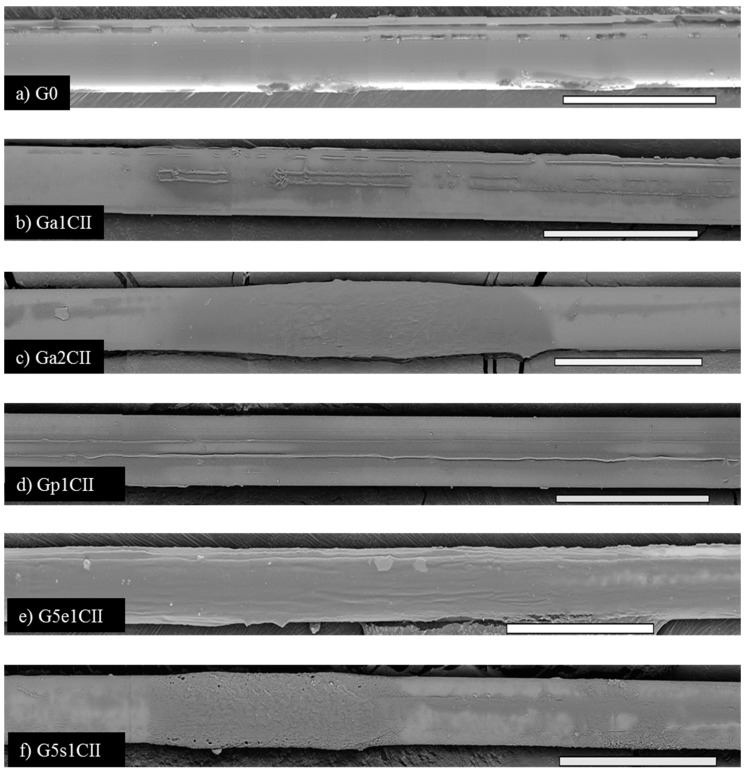
Coating morphology observed by SEM for different coating dispersions. (**a**) Control G0, and various coatings containing CNCPh: (**b**) Ga1CII, (**c**) Ga2CII, (**d**) Gp1CII, (**e**) G5e1CII, (**f**) G5s1CII. Scale bar = 50 µm.

Representative images of the surface of the as-received GF and as-received GF with additional coatings are shown in Figure 4. The coatings on the GF surface are observed as streak lines, films, and regions in darker shades. In comparison to the as-received GF in Figure 4a, coated GF showed an increase in dark regions. Figure 4b–f are representative of the aqueous only CNC coating, aqueous PLL-CNC coating, laboratory emulsion-CNC coating, and commercial emulsion-CNC coating, respectively. Aqueous coatings of CNCs in Figure 4b,d, Ga1CII and Gp1CII, showed a similar rectangular film type coating morphology on the GF surface, with Gp1CII exhibiting better surface coverage. At a higher CNC concentration of 2%, thicker regions of coatings were observed (Figure 4c, Ga2CII). Figure 4e,f is representative of laboratory emulsion and commercial emulsion coatings, respectively. Emulsion coatings showed a thicker coating morphology and more surface coverage than aqueous coatings. Laboratory prepared emulsions displayed superior coating homogeneity to commercial emulsions.

While quantitative comparison of the fiber coatings was not possible using the SEM data, thermogravimetric analysis was used to determine the mass percentage of coatings on the GF surface for various formulations. Dip coated chopped fiber bundles were analyzed from room temperature to 600 °C at 10 °C/min in a nitrogen atmosphere. The coating content of as-received GF was 0.75%, however, the aqueous-CNC and emulsion-CNC formulations coating content increased to 2.15% and 7.44%, respectively, as shown in Table 3.

### 3.3. Fragmentation Behavior

**Figure 5:**
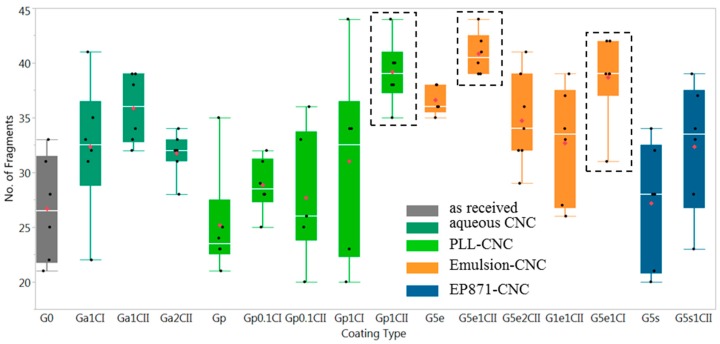
Comparison of the number of fragments observed from the SFFT for the different coating formulations on the GF. Box plot colors indicate different coating media. Grey (G: as received GF), green (a: aqueous only), bright green (p: aqueous with PLL), orange (e: emulsion), and blue (s: commercial emulsion). Box plots enclosed by dotted rectangles are coating formulations with a statistically significant difference in the mean number of fragments compared to G0.

The number of fragments measured for each sample following the SFFT is presented in Figure 5. The results are grouped into five categories depending on the dispersing medium used in the coatings; each category is shown using a different color. The first category (grey) refers to the as-received GF and provides the control/reference case, whereas the other categories consist of the GF that has been coated with aqueous (green), aqueous with PLL (bright green), laboratory epoxy emulsion (orange), and EP871 commercial emulsion (blue) dispersions. Within each category, the effects of the CNC type and CNC concentration in the dispersion were also investigated. In Figure 5, each black dot represents a single test result and the solid-colored rectangles indicate interquartile ranges, the “middle 50%” of data between the 25th and 75th percentiles. The median and mean fragment number values are represented by the line that divides each rectangle and red diamond, respectively. The higher scatter of the fragment number values for some coating formulations could be attributed to coating inhomogeneity.

Several coating formulations showed higher average fragmentation than G0, suggesting improved interfacial interactions over the control. In order to verify the statistical significance of these results, the Tukey–Kramer mean value comparison using JMP Pro 12 software [35] was implemented using a *p*-value criterion of *p* < 0.01. The coating formulations that showed a significant increase in fragmentation over G0 are displayed in the dashed rectangular boxes in Figure 5. These coatings are Gp1CII (GF coat 0.01% PLL 1% CNCPh), G5e1CII (GF coat 5% Emulsion 1% CNCPh), and G5e1CI (GF coat 5% Emulsion 1% CNCS).

### 3.4. Photoelastic Features at Fragmentation

**Figure 6:**
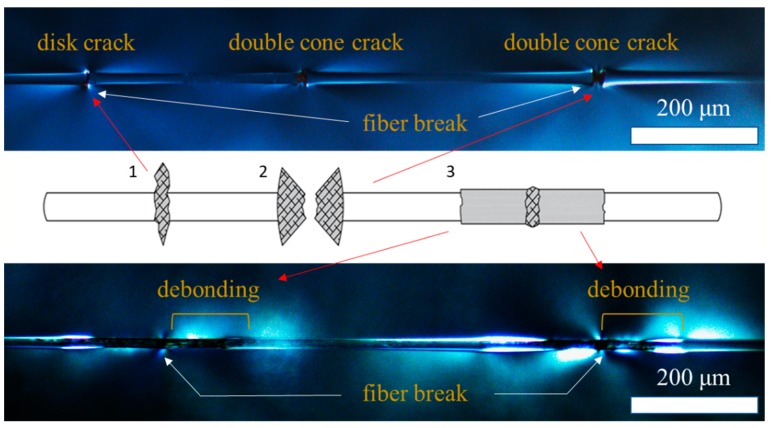
Interfacial fracture modes observed in single fiber fragmentation. (1) Disc-shaped matrix cracking indicates strong interface. (2) Double cone matrix crack represents intermediate interface strength. (3) Matrix debonding along the surface of the GF indicates a weak interface.

Birefringence patterns around the fragmented regions were observed using cross-polarized optical microscopy. The birefringence occurs on either side of the fiber breaking point due to the high shear stress originating during the fiber break and consecutive plastic deformation of the surrounding matrix, which relates to its interfacial properties. Once tensile testing is complete, the remaining birefringence patterns may be visible due to residual stress in the epoxy matrix. There are three distinct fiber breaking zone types, shown schematically in Figure 6: (1) Disk-shaped, which indicates a matrix crack perpendicular to the fiber break and represents a strong interface relative to the strength of the matrix; (2) double cone-shaped cracks near the fiber, indicative of intermediate strength or a strong interface with a low shear strength matrix; and (3) more extensive birefringence patterns with debonding of the matrix from the fiber initiating from the breaking point, which indicates a weak interface [36,37,38].

**Figure 7:**
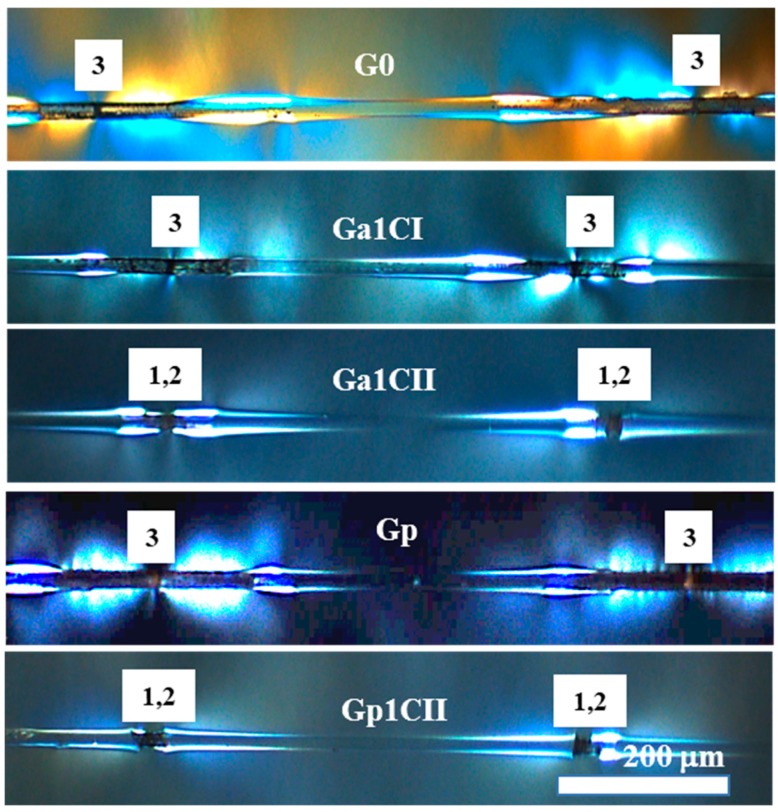
The photoelastic effects of the SFFT for aqueous-based coating formulation samples: G0, Ga1CI, Ga1CII, Gp, and Gp1CII coating formulations.

**Figure 8:**
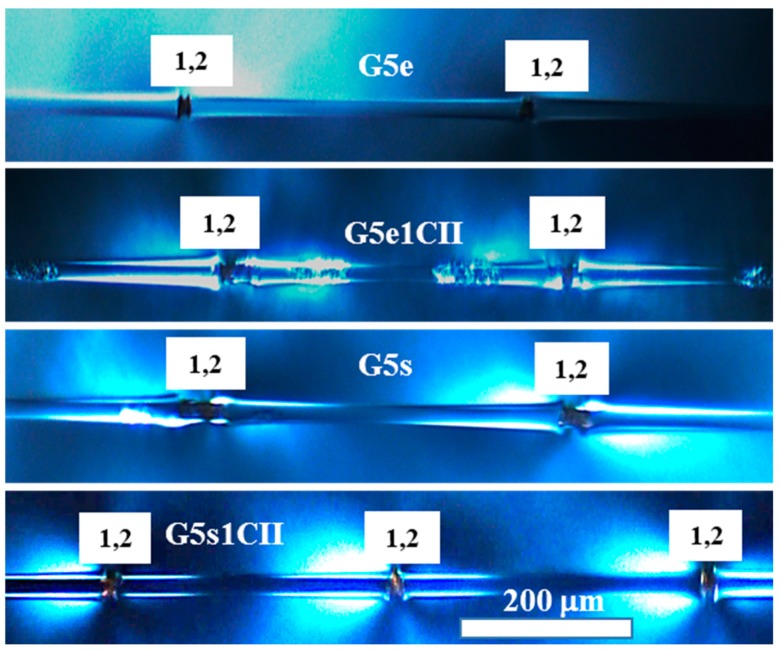
The photoelastic effects of the SFFT for epoxy-based coating formulation samples: G5e, G5e1CII, G5s, and G5S1CII.

Representative fracture modes of GF coated with select formulations are shown in Figure 7 and Figure 8. As shown in Figure 7, G0 (the as-received GF used as a control) and Gp (GF coated with 0.01% PLL solution) exhibited extended debonding, corresponding to the third fracture type and indicating weak fiber–polymer interactions. By comparison, Ga1CI (GF coated with 1% aqueous CNCS) showed a somewhat shorter debonding length by up to 100 µm (length of debonding zone on either side of fiber break); it still primarily exhibited type 3 behavior. Given that no such change was seen in Gp, this indicates that the introduction of CNCS influences the fiber–polymer interphase, though the effect was not sufficient to produce a statistically significant increase in the fragment count. More significantly, the failure modes for Ga1CII and Gp1CII, where the GFs were coated with CNCPh, were changed to a hybrid of the types 1 and 2, with essentially no debonding.

A comparison of the photoelastic fracture patterns for the GF coated with CNCs in the emulsion and commercial sizing is given in Figure 8. A hybrid of type 1 and 2 was dominant, though G5s exhibited more type 3 fractures than G5e, G5e1CII, and G5sCII.

## 4. Discussion

### 4.1. Effect of Dispersion Media on Fragmentation

The effect of the coating dispersion media on the number of fragments obtained from the SFFT can be inferred from Figure 5. Among the coatings with a 1% concentration of CNCs, the laboratory emulsion coatings (G5e1CI, G5e1CII) and the 0.01% PLL coating with 1% CNCPh (Gp1CII) exhibited the highest average fragmentation. Such high fragmentation suggests high interfacial adhesion; this result was supported by the superior surface coverage and homogeneity G5e1CII and Gp1CII displayed in Figure 4d,e. Further support was provided in Figure 7 and Figure 8 by the predominance of fracture types 1 and 2. In comparison to the as-received GF (G0), the mean fragmentation lengths for these samples increased by 45% to 53%, a statistically significant fragmentation length increase according to the Tukey–Kramer method (*p* < 0.01) [35].

In the case of aqueous PLL coatings, this increase was only observed upon incorporation of CNCs. The presence of PLL on the glass surface appears to slightly weaken the adhesion to the epoxy, resulting in extended debonding (Figure 7, Gp), scattering in the fragmentation data, and a slight decrease in the mean fragmentation number (Figure 5, G0 versus Gp). However, for PLL coatings with CNCPh, the fragmentation number increased compared to its aqueous-only analogue. This suggests that PLL may play a role in improving the distribution or adhesion of CNCPh to the fiber surface, which may be related to its ability to alter the surface charge [39].

CNCs had a similar effect on the fragmentation number in commercial emulsions, resulting in an increased average number of fragments for G5s1CII relative to the emulsion-only control, G5s, as shown in Figure 5. The same was true for the laboratory emulsions, G5e1CII and G5e1CI, when compared to G5e. When comparing fragmentation counts for G5e1CII and G5e1CI to the as-received fiber, G0, a statistically significant increase in the fragmentation number was found (*p* < 0.01); however, when comparing these lab emulsion-CNC coatings to each other, no statistically significant increase was found. Moreover, the average fragmentation of aqueous CNCPh and CNCS coatings (Ga1CII, Ga1CI) was statistically similar to their emulsion-based counterparts. Determining the cause of this behavior requires a better understanding of how CNC-only and CNC-emulsion systems affect the interphase. Based on the differences in fracture type observed between Figure 7 and Figure 8, it is possible that the presence of epoxy and polymer surfactants in the laboratory and commercial emulsion systems, respectively, results in interphase toughening, leading to fracture types 1 and 2 with minimal debonding. From the results in Figure 5, the presence of CNCs may impact this toughening phenomenon. In the case of CNC-only systems, the CNCs on the as-received GF surface may moderately toughen the matrix epoxy at the interface, while overcoating and encapsulation of CNCs in the emulsion-CNC systems may further increase CNC–GF and CNC–matrix interactions.

### 4.2. Effect of CNC Surface Functionalization on Fragmentation

The CNC functionalizations studied were CNCS and CNCPh. At a 1% concentration, CNCPh outperformed CNCS in all dispersion media in the average fragment count and data scatter, as shown in Figure 5. In the PLL coatings, fragmentation increased over G0 by 48% for CNCPh (Gp1CII) and 16% for CNCS (Gp1CI). In the laboratory emulsions (G5e1CII and G5e1CI), these values were 52% and 44%, respectively. These improvements may be related to the methyl(triphenyl) phosphonium modification of CNCPh; as previously reported, it reduces CNC–CNC interactions and water absorption, leading to less agglomeration and an increased CNC–epoxy interfacial area [24]. This is further reflected in the fracture patterns in Figure 7, with the mixture of type 1 and 2 fractures exhibited by Ga1CII supporting better interaction with the epoxy matrix and a stronger interphase than Ga1CI.

CNC interactions with the emulsion system and GF were probed by FTIR, showing similar peak shifts in Figure 2 and Figure 3. The OH peak at 3331 cm^−1^, indicative of intermolecular hydrogen bonding in pristine CNCS and CNCPh, shifted to 3339 cm^−1^. This blue shift in the presence of the GF and the emulsion could be attributed to the disruption of native hydrogen bonds, lower availability of hydrogen bond free hydroxyl groups, or interaction with a new phase, as others have previously shown [33,40]. The blue shift of the C-O group from 1053 to 1058 cm^−1^ could also be attributed to the interaction of CNCS and CNCPh with the emulsion system. Thus, neither CNC type exhibited preferential interactions for the GF or emulsion.

### 4.3. Effect of CNC Concentration on Fragmentation

The concentrations of CNCs and laboratory emulsions in the coating formulations showed a significant effect on fragmentation. For the PLL-based coatings, 0.1% concentrations of CNCPh and CNCS failed to improve the average fragmentation over G0 due to the minimal presence of CNCs on the GF surface. However, a significant increase in fragmentation was observed when the concentration was increased to 1%. Laboratory emulsions also showed greater fragmentation at higher concentrations (i.e., 5% vs. 1%) regardless of the presence of CNCs (G5e1CII vs. G1e1CII, Figure 5). Thus, it can be inferred from the current formulations that a minimum of 5% laboratory emulsion or 5% EP871 is required to improve GF–matrix adhesion.

Further increases in the CNC concentration from 1% to 2% did not show improvements in the mean fragmentation. For CNCPh aqueous and emulsion coatings, fragmentation was reduced with increasing CNC concentration. Figure 4c shows that this higher concentration of CNCPh led to random thick coating films, indicating an increase in the aggregation of CNCPh at the GF–matrix interface. As the CNC concentration was the only parameter varied in this comparison, this suggests that aggregation may limit the interface strength with the epoxy, thus resulting in the reduction in fragmentation observed in Figure 5. Moreover, higher concentrations of CNCs also increase CNC aggregation in coating dispersions, which may affect coating homogeneity. These results suggest that 1% is the optimized CNC concentration for both CNC types in all formulations.

## 5. Conclusions

This work investigated the potential of CNCs to enhance the interfacial shear strength in GF–epoxy composites when used in the GF coating/sizing. Different CNC types, dispersion concentrations, and coating formulations, including aqueous systems, polymer emulsions, and commercial emulsions, were studied. In addition to the interfacial adhesion determined by the SFFT, the fracture type of the fiber inside the epoxy under tensile loading, as observed by cross-polarized microscopy, indicated that CNCs enhance GF–epoxy interactions.

Specifically, based on Tukey–Kramer statistical analysis for *p* < 0.01, it can be concluded that certain coating formulations containing 1% CNCs (Gp1CII, G5e1CII, and Ge1CI) resulted in significant increases in the fragmentation count as compared to the as-received GF. Even with a broader *p*-value range (*p* < 0.05), the same three formulations were the only ones to show statistical significance. However, a notable interface enhancement with higher fragmentation as compared to G0 was observed for other aqueous and emulsion coatings. Among these formulations, increases in the average fragmentation and changes in fracture patterns were observed for a minimum CNC content of 1% in aqueous coatings. Moreover, the addition of 1% CNCs in emulsion coatings (laboratory/commercial) increased the fragmentation number relative to emulsion-only coatings. As for CNC surface functionality, although statistically similar by the Tukey–Kramer analysis, the interface enhancement was higher for CNCPh than CNCS for all formulations. Finally, it can be concluded that CNC-only coatings exhibit a combination of type 3 fractures and hybrid type 1 and 2 fractures with less debonding and birefringence than as-received GF, and fracture types 1 and 2 are dominantly observed in emulsion media coatings.

In summary, it was shown that the addition of CNCs in the sizing solution of glass fibers can lead to stronger fiber–epoxy interactions, which indicates the potential of CNCs as a sizing component for glass fiber manufacturing. The presence of a nanofiller on the fiber probably improves adhesion through several mechanisms, which may include interphase toughening with interpenetrating network formation and alterations of the microstructure by the nanofiller [41,42], increased interphase debonding resistance along the fiber length [42,43], increased surface roughness [15], increased fiber bridging into the matrix [44], and bond formation with the fiber and matrix [42,45]. In the case of CNC-based coating, several of these mechanisms can contribute to better adhesion, such as the presence of hydroxyl groups on the CNCs that can interact with the GF surface and with epoxide rings in the epoxy [46], or CNC microstructure impregnation with cured epoxy that can toughen the interphase and resist matrix interphase debonding. Thus, further studies are required to elucidate the mechanism behind the increase of the interfacial shear strength by CNCs.

## Figures and Tables

**Table 1:** Details of the naming scheme of the coating formulations.

	1st Part	2nd Part	3rd Part	4th Part	5th Part
**Variable**:	Fiber type	Dispersant concentration%	Dispersion medium	CNC% in solution	CNC functionality
**Values**:	G (as-received fibers)	1 (emulsions) 5 (emulsions) N/A (aqueous)	a (aqueous) p (0.01% PLL in water, pH 8) e (laboratory emulsion) s (commercial emulsion)	0.1 1 2	CI (sodium sulfate) CII (methyl (triphenyl) phosphonium)

**Table 2:** Coating preparations and their respective labels.

Sample	Coating Type	Sample	Coating Type
**G0**	As-received	**G5e**	GF coat 5%Emulsion
**Ga1CI**	GF coat 1%CNCS	**G5e1CII**	GF coat 5%Emulsion 1%CNCPh
**Ga1CII**	GF coat 1%CNCPh	**G5e2CII**	GF coat 5%Emulsion 2%CNCPh
**Ga2CII**	GF coat 2%CNCPh	**G1e1CII**	GF coat 1%Emulsion 1%CNCPh
**Gp**	GF coat 0.01%PLL	**G5e1CI**	GF coat 5%Emulsion 1%CNCS
**Gp0.1CI**	GF coat 0.01%PLL 0.1%CNCS	**G5s**	GF coat 5%EP871
**Gp0.1CII**	GF coat 0.01%PLL 0.1%CNCPh	**G5s1CII**	GF coat 5%EP871 1%CNCPh
**Gp1CI**	GF coat 0.01%PLL 1%CNCS		
**Gp1CII**	GF coat 0.01%PLL 1%CNCPh		

**Table 3:** Mass percent (%) of coating for select formulations (parentheses indicate one standard deviation).

Coating Type	G0	Ga1CII	G5e	G5e1CII	G5s1CII
Avg. mass%	0.75 (0.03)	2.15 (0.38)	3.92 (0.13)	6.66 (0.71)	7.44 (0.91)

## Disclaimer

Research carried out at the National Institute of Standards and Technology (NIST), an agency of the U. S. government and by statute is not subject to copyright in the United States. Certain commercial equipment, instruments, materials or companies are identified in this paper in order to adequately specify the experimental procedure. Such identification is not intended to imply recommendation or endorsement by the National Institute of Standards and Technology, nor is it intended to imply that the materials or equipment identified are necessarily the best available for this purpose.

## References

[1] Gattesco N., Amadio C., Bedon C. (2015). Experimental and numerical study on the shear behavior of stone masonry walls strengthened with GFRP reinforced mortar coating and steel-cord reinforced repointing. Eng. Struct..

[2] Valluzzi M.R., Modena C., de Felice G. (2014). Current practice and open issues in strengthening historical buildings with composites. Mater. Struct..

[3] Siwowski T., Rajchel M., Kaleta D., Własak L. (2017). The First Polish Road Bridge Made of FRP Composites. Struct. Eng. Int..

[4] Bedon C., Agullo C.P., Luna-Navarro A., Overend M., Favoino F. (2018). Thermo-mechanical Investigation of Novel GFRP-glass Sandwich Facade Components. Chall. Glass Conf. Proc..

[5] Friedrich K., Almajid A.A. (2013). Manufacturing Aspects of Advanced Polymer Composites for Automotive Applications. Appl. Compos. Mater..

[6] Gu Y., Li M., Wang J., Zhang Z. (2010). Characterization of the interphase in carbon fiber/polymer composites using a nanoscale dynamic mechanical imaging technique. Carbon.

[7] Hodzic A., Stachurski Z.H., Kim J.K. (2000). Nano-indentation of polymer–glass interfaces Part I. Experimental and mechanical analysis. Polymer.

[8] Thomason J.L., Adzima L.J. (2001). Sizing up the interphase: An insider’s guide to the science of sizing. Compos. Part A Appl. Sci. Manuf..

[9] Mäder E., Gao S.L., Plonka R. (2007). Static and dynamic properties of single and multi-fiber/epoxy composites modified by sizings. Compos. Sci. Technol..

[10] Thomason J.L. (1995). The interface region in glass fibre-reinforced epoxy resin composites: 3. Characterization of fibre surface coatings and the interphase. Composites.

[11] Zhang J., Zhuang R., Liu J., Mäder E., Heinrich G., Gao S. (2010). Functional interphases with multi-walled carbon nanotubes in glass fibre/epoxy composites. Carbon.

[12] Zinck P., Mäder E., Gerard J.F. (2001). Role of silane coupling agent and polymeric film former for tailoring glass fiber sizings from tensile strength measurements. J. Mater. Sci..

[13] Mahmood H., Tripathi M., Pugno N., Pegoretti A. (2016). Enhancement of interfacial adhesion in glass fiber/epoxy composites by electrophoretic deposition of graphene oxide on glass fibers. Compos. Sci. Technol..

[14] Qin W., Vautard F., Drzal L.T., Yu J. (2015). Mechanical and electrical properties of carbon fiber composites with incorporation of graphene nanoplatelets at the fiber–matrix interphase. Compos. Part B Eng..

[15] Gao X., Jensen R.E., McKnight S.H., Gillespie J.W. (2011). Effect of colloidal silica on the strength and energy absorption of glass fiber/epoxy interphases. Compos. Part A Appl. Sci. Manuf..

[16] Moon R.J., Martini A., Nairn J., Simonsen J., Youngblood J. (2011). Cellulose nanomaterials review: Structure, properties and nanocomposites. Chem. Soc. Rev..

[17] Brinchi L., Cotana F., Fortunati E., Kenny J.M. (2013). Production of nanocrystalline cellulose from lignocellulosic biomass: Technology and applications. Carbohydr. Polym..

[18] Moon R.J., Schueneman G.T., Simonsen J. (2016). Overview of Cellulose Nanomaterials, Their Capabilities and Applications. JOM.

[19] Börjesson M., Westman G. (2015). Crystalline nanocellulose—Preparation, modification, and properties. Cellulose-Fundamental Aspects and Current Trends.

[20] Lu P., Hsieh Y.L. (2010). Preparation and properties of cellulose nanocrystals: Rods, spheres, and network. Carbohydr. Polym..

[21] Habibi Y. (2014). Key advances in the chemical modification of nanocelluloses. Chem. Soc. Rev..

[22] De Assis C.A., Houtman C., Phillips R., Bilek E., Rojas O.J., Pal L., Peresin M.S., Jameel H., Gonzalez R. (2017). Conversion Economics of Forest Biomaterials: Risk and Financial Analysis of CNC Manufacturing. Biofuels Bioprod. Biorefining.

[23] Chen L., Zhu J.Y., Baez C., Kitin P., Elder T. (2016). Highly thermal-stable and functional cellulose nanocrystals and nanofibrils produced using fully recyclable organic acids. Green Chem..

[24] Fox D.M., Rodriguez R.S., Devilbiss M.N., Woodcock J., Davis C.S., Sinko R., Keten S., Gilman J.W. (2016). Simultaneously Tailoring Surface Energies and Thermal Stabilities of Cellulose Nanocrystals Using Ion Exchange: Effects on Polymer Composite Properties for Transportation, Infrastructure, and Renewable Energy Applications. ACS Appl. Mater. Interfaces.

[25] Asadi A., Miller M., Moon R., Kalaitzidou K. (2016). Improving the interfacial and mechanical properties of short glass fiber/epoxy composites by coating the glass fibers with cellulose nanocrystals. Express Polym. Lett..

[26] Chen Y., Zhou X., Yin X., Lin Q., Zhu M. (2014). A Novel Route to Modify the Interface of Glass Fiber-Reinforced Epoxy Resin Composite via Bacterial Cellulose. Int. J. Polym. Mater. Polym. Biomater..

[27] Reid M.S., Villalobos M., Cranston E.D. (2017). Benchmarking Cellulose Nanocrystals: From the Laboratory to Industrial Production. Langmuir.

[28] Kelly A., Tyson W.R. (1965). Tensile properties of fibre-reinforced metals: Copper/tungsten and copper/molybdenum. J. Mech. Phys. Solids.

[29] Holmes G.A., Peterson R.C., Hunston D.L., McDonough W.G., Schutte C.L., Schapery R.A. (2000). The Effect of Nonlinear Viscoelasticity on Interfacial Shear Strength Measurements. Time Dependent and Nonlinear Effects in Polymers and Composites.

[30] Hui C.Y., Phoenix S., Shia D. (1998). The single-filament-composite test: A new statistical theory for estimating the interfacial shear strength and Weibull parameters for fiber strength. Compos. Sci. Technol..

[31] Zhandarov S., Pisanova E., Dovgyalo V. (1992). Fragmentation of a single filament during tension in a matrix as a method of determining adhesion. Mech. Compos. Mater..

[32] Kargarzadeh H., MSheltami R., Ahmad I., Abdullah I., Dufresne A. (2015). Cellulose nanocrystal: A promising toughening agent for unsaturated polyester nanocomposite. Polymer.

[33] Xu X., Liu F., Jiang L., Zhu J.Y., Haagenson D., Wiesenborn D.P. (2013). Cellulose Nanocrystals vs. Cellulose Nanofibrils: A Comparative Study on Their Microstructures and Effects as Polymer Reinforcing Agents. ACS Appl. Mater. Interfaces.

[34] Stoch L., Środa M. (1999). Infrared spectroscopy in the investigation of oxide glasses structure. J. Mol. Struct..

[35] (2015). Basic Analysis. JMP^®^ 12 Documentation Library.

[36] Drzal L.T., Madhukar M. (1993). Fibre-matrix adhesion and its relationship to composite mechanical properties. J. Mater. Sci..

[37] Kim B.W., Nairn J.A. (2002). Observations of Fiber Fracture and Interfacial Debonding Phenomena Using the Fragmentation Test in Single Fiber Composites. J. Compos. Mater..

[38] Zhou X.F., Nairn J.A., Wagner H.D. (1999). Fiber–matrix adhesion from the single-fiber composite test: Nucleation of interfacial debonding. Compos. Part A Appl. Sci. Manuf..

[39] Michael T.P., András V., John D., Natalia F., Bin M., Ryan W., Arvind R., Robert J.M., Ronald S., Theodore H.W. (2011). Development of the metrology and imaging of cellulose nanocrystals. Meas. Sci. Technol..

[40] Mo Z.L., Zhao Z.L., Chen H., Niu G.P., Shi H.F. (2009). Heterogeneous preparation of cellulose-polyaniline conductive composites with cellulose activated by acids and its electrical properties. Carbohydr. Polym..

[41] Zhang X., Fan X., Yan C., Li H., Zhu Y., Li X., Yu L. (2012). Interfacial microstructure and properties of carbon fiber composites modified with graphene oxide. ACS Appl. Mater. Interfaces.

[42] Karger-Kocsis J., Mahmood H., Pegoretti A. (2015). Recent advances in fiber/matrix interphase engineering for polymer composites. Prog. Mater. Sci..

[43] Li Q., Church J.S., Naebe M., Fox B.L. (2016). Interfacial characterization and reinforcing mechanism of novel carbon nanotube—Carbon fibre hybrid composites. Carbon.

[44] Zhu J., Imam A., Crane R., Lozano K., Khabashesku V.N., Barrera E.V. (2007). Processing a glass fiber reinforced vinyl ester composite with nanotube enhancement of interlaminar shear strength. Compos. Sci. Technol..

[45] Gao S.L., Mäder E., Plonka R. (2008). Nanocomposite coatings for healing surface defects of glass fibers and improving interfacial adhesion. Compos. Sci. Technol..

[46] Ansari F., Galland S., Johansson M., Plummer C.J.G., Berglund L.A. (2014). Cellulose nanofiber network for moisture stable, strong and ductile biocomposites and increased epoxy curing rate. Compos. Part A Appl. Sci. Manuf..

